# An antibacterial from *Hypericum acmosepalum* inhibits ATP-dependent MurE ligase from *Mycobacterium tuberculosis*

**DOI:** 10.1016/j.ijantimicag.2011.09.018

**Published:** 2012-02

**Authors:** Khadijo Osman, Dimitrios Evangelopoulos, Chandrakala Basavannacharya, Antima Gupta, Timothy D. McHugh, Sanjib Bhakta, Simon Gibbons

**Affiliations:** a Department of Pharmaceutical and Biological Chemistry, The School of Pharmacy, University of London, 29–39 Brunswick Square, Bloomsbury, London WC1N 1AX, UK; b Institute of Structural and Molecular Biology, Department of Biological Sciences, Birkbeck, University of London, Malet Street, Bloomsbury, London WC1E 7HX, UK; c Centre for Clinical Microbiology, Department of Infection, Royal Free Campus, University College London, London NW3 2PF, UK

**Keywords:** *Hypericum acmosepalum*, Hyperenone A, Hypercalin B, *Staphylococcus aureus*, Tuberculosis, Peptidoglycan, MurE ligase

## Abstract

In a project to characterise new antibacterial chemotypes from plants, hyperenone A and hypercalin B were isolated from the hexane and chloroform extracts of the aerial parts of *Hypericum acmosepalum.* The structures of both compounds were characterised by extensive one- and two-dimensional nuclear magnetic resonance (NMR) spectroscopy and were confirmed by mass spectrometry. Hyperenone A and hypercalin B exhibited antibacterial activity against multidrug-resistant strains of *Staphylococcus aureus*, with minimum inhibition concentration ranges of 2–128 mg/L and 0.5–128 mg/L, respectively. Hyperenone A also showed growth-inhibitory activity against *Mycobacterium tuberculosis* H37Rv and *Mycobacterium bovis* BCG at 75 mg/L and 100 mg/L. Neither hyperenone A nor hypercalin B inhibited the growth of *Escherichia coli* and both were non-toxic to cultured mammalian macrophage cells. Both compounds were tested for their ability to inhibit the ATP-dependent MurE ligase of *M. tuberculosis*, a crucial enzyme in the cytoplasmic steps of peptidoglycan biosynthesis. Hyperenone A inhibited MurE selectively, whereas hypercalin B did not have any effect on enzyme activity.

## 1 Introduction

In recent years, drug resistance in *Mycobacterium tuberculosis* and other clinically relevant bacterial pathogens such as meticillin-resistant *Staphylococcus aureus* (MRSA) has emerged as a major threat to public health, with severe economic and social implications [1,2]. MRSA is still one of the most widespread and virulent nosocomial pathogens in the world [3] and is usually resistant to multiple antibiotics, making infection difficult to treat, and accounts for an increased proportion of staphylococcal infections amongst hospitalised patients in countries where it has become established. Despite new advances in antibiotic development, with agents such as linezolid, daptomycin and quinupristin/dalfopristin appearing over the last decade [4], MRSA infections still remain of considerable concern owing to resistance to some of these new drugs [5,6].

Tuberculosis (TB) is caused predominantly by *M. tuberculosis*, an obligate aerobic pathogen that divides at an extremely slow rate. The World Health Organization estimates that 2 billion people have latent TB whilst another 2 million people die of TB each year worldwide [7]. Although TB is generally treatable, the exponential emergence of extensively drug-resistant or completely drug-resistant TB strains is a current global health emergency where there is virtually no treatment available [8]. Therefore, new antibacterial agents with novel modes of action are urgently needed to meet the challenge posed by these resistant variants.

The peptidoglycan biosynthesis pathway is a validated target for the development of antibacterial agents, including the classical β-lactam, cephalosporin and glycopeptide antibacterials [9]. The peptidoglycan layer of the cell wall serves as a base for the lipid-rich capsule. Peptidoglycan (or murein) is the polymeric mesh of the bacterial cell wall that plays a vitally important role in protecting bacteria against osmotic lysis. Peptidoglycan biosynthesis can be separated into two phases, comprising six intracellular enzymatic steps and three steps that occur outside of the plasma membrane [10]. Amongst the cytoplasmic steps involved in the biosynthesis of peptidoglycan, four ATP-dependent ligases (MurC, MurD, MurE and MurF) catalyse the assembly of its peptide moiety by successive additions of l-alanine, d-glutamate, a diamino acid [usually *meso*-diaminopimelic acid (*m*-DAP) or l-lysine] and d-alanine–d-alanine to UDP *N*-acetylmuramic acid (MurNAc) [11]. Although most antimicrobials target steps in the later stage of cell wall biosynthesis, the earlier steps in peptidoglycan biosynthesis have recently received renewed attention in terms of novel therapeutic targets [12]. To date, only fosfomycin, which targets MurA (NAG enolpyruvate transferase), has been developed as an antibacterial agent targeting early biosynthesis of cell wall peptidoglycan [10,11]. The enzymes involved in the peptidoglycan biosynthetic pathway are characteristic of Eubacteria and are absent in humans. These enzymes are therefore excellent targets for anti-infective drug development.

Although most antibiotics in clinical use have been obtained from microorganisms, an interest in plant antibacterials has re-emerged during the last three decades. Of the known plant species (estimated at 250 000), only a very small fraction has been investigated for the presence of antibacterial compounds and an even smaller number of these phytochemicals have been subjected to a modern rigorous pharmacological evaluation of their antibacterial properties.

Species of the plant genus *Hypericum* are used in traditional medicine for their therapeutic value [13]. *Hypericum perforatum* L. (commonly known as St John's Wort) is the most investigated member of the genus both from the perspective of chemical constituents and biological activity [14]. It has been used extensively in herbal medicine as an antidepressant [14], for various skin treatments such as eczema, wounds and burns, as well as in disorders of the alimentary tract, amongst others [14].

*Hypericum acmosepalum* is a shrub of 0.6–2 m in height with deep-yellow petals that are sometimes tinged red. It is cultivated as an ornamental shrub in many parts of the world, but particularly in Great Britain [15]. Previous studies on *H. acmosepalum* are limited, with some ethnobotanical usage by the Yao People of Yunnan Province in China [16]. Here we describe the isolation, structural elucidation and antibacterial action of two natural products from *H. acmosepalum*. Their ability to inhibit the ATP-dependent MurE ligase enzyme from *M. tuberculosis* [10], a pivotal enzyme in peptidoglycan biosynthesis, was also assessed.

## 2 Materials and methods

### 2.1 Bacterial strains

A standard *S*. *aureus* strain (ATCC 25923) as well as a clinical isolate (XU212) that possesses the Tet(K) efflux pump and is also an MRSA strain were obtained from Dr Edet Udo (Kuwait University) [17]. Epidemic MRSA types 15 and 16 (EMRSA-15 and EMRSA-16) were provided by Dr Paul Stapleton (The School of Pharmacy, University of London, UK). Strain SA-1199B that overexpresses the NorA multidrug resistance efflux pump was donated by Prof. Glenn W. Kaatz (Wayne State University, Detroit, MI) [18]. Strain RN4220 that has the MsrA macrolide efflux pump was a generous gift from Dr Jon Cove (University of Leeds, UK) [19]. *Mycobacterium bovis* BCG (ATCC 35734) and RAW 264.7 (a mouse leukaemic monocyte macrophage cell line) were obtained from Prof. Siamon Gordon (Sir William Dunn School of Pathology, University of Oxford, Oxford, UK). *Mycobacterium tuberculosis* H37Rv (ATCC 9360) was purchased from the Health Protection Agency (Porton Down, Salisbury, UK).

### 2.2 Isolation of compounds

*Hypericum acmosepalum* was collected from the National *Hypericum* Collection at the Royal Botanic Gardens, Kew (Ardingly, UK) in August 2005. The authenticity of this species has been verified by Dr N.K.B. Robson. Voucher specimens of these collections have been deposited at the Department of Pharmaceutical and Biological Chemistry (SG-2005-2/6) (School of Pharmacy, University of London, London, UK). Dried and powdered material (500 g) of *H. acmosepalum* was extracted sequentially in a Soxhlet apparatus (Fisher Scientific, Loughborough, UK) using 3.5 L of organic solvents of increasing polarity (hexane, chloroform and methanol). Vacuum-liquid chromatography (VLC) (Silica gel 60 PF_254+366_; Merck, Darmstadt, Germany) was performed on 6.3 g of the hexane extract with an increasingly polar gradient of 10% increments, from 100% hexane to 100% ethyl acetate, yielding 12 fractions. Thin-layer chromatography (TLC) of Fraction 6 showed a major compound that was subjected to LH-20 Sephadex column chromatography (Sigma Aldrich, Gillingham, UK) to give 12 fractions by elution with hexane, chloroform and chloroform–methanol mixtures. Fractions 6, 7 and 8 eluted with 30% hexane and in chloroform were combined yielding pure compound **1** (94 mg). The chloroform extract (8.3 g) was fractionated by VLC with an increasingly polar gradient of 10% from 100% hexane to 100% ethyl acetate, yielding 12 fractions. Fractions 6 and 7 showing a similar TLC profile were combined (2.5 g) and were further separated using Sephadex column chromatography using the same method described above. Fractions 2 and 3 eluted with 35% hexane in chloroform from Sephadex were combined (160 mg) and further separated by preparative TLC silica plates (20 mm × 20 mm, 60F_254_; Merck) using hexane–ethyl acetate–acetic acid (85:15:2) to give compound **2** (25 mg).

#### 2.2.1 Compound **1** (hypercalin B)

Pale yellow amorphous solid. Electrospray ionisation mass spectrometry (ESI-MS) [M+H]^+^ *m*/*z* 519, calculated for C_33_H_43_O_5_. Ultraviolet (UV) (CHCl_3_) *λ*_max_ (log *ɛ*): 360 (4.09), 285 (3.56), 244 (3.81) nm. Infrared (IR) vapour maximum (*V*_max_ thin film) cm^−1^: (3370, 3060, 2960, 2920, 28 260). Proton (^1^H) and carbon (^13^C) nuclear magnetic resonance (NMR) spectroscopic data were in close agreement with those of hypercalin B isolated from *Hypericum calycinum* [20].

#### 2.2.2 Compound **2** (hyperenone A)

Yellow oil. High-resolution ESI-MS [M+H]^+^ *m*/*z* 271.1396 calculated for C_17_H_19_O_3._ UV (CHCl_3_) *λ*_max_ (log *ɛ*): 236 (4.12), 276 (4.07) nm. IR *V*_max_ (thin film) cm^−1^: (1651, 1614, 1580). ^1^H and ^13^C NMR spectroscopic data (Table 1).

### 2.3 Structure elucidation

One-dimensional (1D) and two-dimensional (2D) NMR spectra were recorded on an Avance 500 MHz spectrometer (Bruker, Coventry, UK). Chemical shift values (*δ*) were reported in parts per million (ppm). Samples were dissolved in deuterated chloroform. IR spectra were recorded on a Nicolet 360 FT-IR spectrophotometer (Thermo Fisher Scientific, Loughborough, UK), and UV spectra on a Thermo Electron Corporation Helios spectrophotometer (Thermo Fisher Scientific). Mass spectra were recorded on a Micromass Q-TOF Global Tandem Mass Spectrometer (Waters Corp., Manchester, UK).

### 2.4 Minimum inhibitory concentration (MIC) determination against *Staphylococcus aureus*

Prior to antibacterial bioassay, strains were subcultured freshly on Mueller–Hinton agar medium (Oxoid Ltd., Basingstoke, UK) and incubated for 24 h at 37 °C prior to MIC determination. Control antibiotics norfloxacin, tetracycline, vancomycin, erythromycin and ciprofloxacin were also used. Mueller–Hinton broth (Oxoid Ltd.) was adjusted to contain 20 mg/L Ca^2+^ and 10 mg/L Mg^2+^. An inoculum density of 5 × 10^5^ colony-forming units/mL of each *S. aureus* strain was prepared in normal saline (9 g/L) by comparison with a 0.5 McFarland turbidity standard. The inoculum (125 μL) was added to all wells of a microtitre plate and the plate was incubated at 37 °C for 18 h. For MIC determination, 20 μL of a 5 mg/mL methanolic solution of 3-(4,5-dimethylthiazol-2-yl)-2,5-diphenyltetrazolium bromide (MTT) was added to each of the wells and was incubated for 20 min. Bacterial growth was indicated by a colour change from yellow to dark blue. The MIC was recorded as the lowest concentration at which no growth was observed.

### 2.5 Minimum inhibitory concentration determination against mycobacteria

*Mycobacterium bovis* BCG was grown at 37 °C in an incubator in 100 mL of Middlebrook 7H9 broth medium supplemented with 10% (v/v) albumin–dextrose–catalase (Difco, Oxford, UK) and 0.05% Tween 80 in roller bottles with rotation at 2 rpm until mid-exponential phase [optical density at 600 nm (OD_600_) = 1.0]. *Mycobacterium tuberculosis* H37Rv was grown at 37 °C in an incubator as a standing culture in 30 mL universals in Middlebrook 7H9 broth medium supplemented with 10% (v/v) oleic acid–albumin–dextrose–catalase (OADC) (Difco) and 0.05% Tween 80 until mid-exponential phase (1 McFarland turbidity standard).

For quality control of the mycobacterial cultures, *M. bovis* BCG was stained with a modified Ziehl–Neelsen staining protocol using a Tb-color kit (Bund Deutscher Hebammen Laboratory, Karlsruhe, Germany) according to the manufacturer's procedure, and *M. tuberculosis* H37Rv was grown on a blood agar plate to detect contamination. MICs against mycobacteria were determined using the spot culture growth inhibition assay as described previously [21]. An aliquot of 5 mL of Middlebrook 7H10 (BD Biosciences, Oxford, UK) supplemented with 0.2% glycerol and 10% OADC was added to a six-well plate along with compounds at concentrations of 100, 75, 50, 25, 10 and 0 mg/L in a 5 μL solution of dimethyl sulphoxide (DMSO). Afterwards, 5 μL of an appropriately diluted mid-log phase of 10^5^ cells/mL was carefully dispensed into the centre of each well. A well with no compound containing 0.1% DMSO was used as a negative control. The MIC was determined after 2 weeks of incubation at 37 °C. A frontline antitubercular drug, isoniazid (INH), was also used as a positive control.

### 2.6 *Escherichia coli* JM109 killing curves

A seed culture of *E. coli* JM109 was grown up to mid-log phase (OD_600_ = 1.9). Then, 1 mL of the seed culture was mixed with 100 mL of fresh Luria–Bertani medium and 5 mL was distributed into 25-mL volume glass culture tubes (Brand GmbH, Wertheim, Germany) containing either the inhibitors used in this study (final concentration 200 mg/L) or DMSO (final concentration 0.1%). These cultures were then grown at 37 °C in a shaking incubator rotating at 180 rpm. The glass tubes were placed into the cell holder of a spectrophotometer (Biochrom WPA CO8000; Biochrom, Cambridge, UK) and the OD_600_ was measured every 30 min until the culture reached stationary phase. INH was taken as a positive control and the experiment was performed in triplicate.

### 2.7 Cytotoxicity assay

Mouse macrophage cells (RAW 264.7) were cultured in 5 mL of complete RPMI 1640 medium supplemented with 10% foetal bovine serum and 1% l-glutamine in a 25 cm^2^ vented, screw-cap cell culture flask (Flowgen Bioscience Ltd., Hessle, UK) and incubated at 37 °C with a supply of 5% CO_2_. For use in cytotoxicity assay, cells were detached using 5 mL of lidocaine–ethylene diamine tetra-acetic acid (EDTA) for 10 min at room temperature, followed by banging the side of the flask against the palm of the hand, and diluted with an equal volume of fresh media. Cells were then centrifuged at 1000 rpm for 5 min at room temperature, washed twice with 1× phosphate-buffered saline, re-suspended in complete RPMI media, counted and diluted to adjust to 10^5^ cells/mL. Then, 100 μL of cells were seeded to each well of a 96-well plate containing final concentrations (3.125–200 mg/L) of inhibitors and controls that had been pre-plated by serial dilution [22]. DMSO and INH were used as negative and positive controls, respectively, and the experiment was performed in triplicate. Following 48 h of incubation at 37 °C in 5% CO_2_, medium was replaced and supplemented with 30 μL of freshly prepared 0.01% resazurin solution. A change in colour from blue to pink indicating the viability of cells was observed after 16 h incubation at 37 °C in 5% CO_2_. For quantitative analysis, the fluorescence intensity was measured by excitation at 560 nm and emission at 590 nm using a fluorescence microtitre plate reader (BMG Labtech GmbH, Ortenberg, Germany).

### 2.8 Inhibition of ATP-dependent MurE ligase activity from *Mycobacterium tuberculosis*

The MurE enzyme activity assay [11] was set up using 100 ng of MurE enzyme in the presence of 25 mM Bis–Tris–propane/HCl (pH 8.5), 5 mM MgCl_2_, 250 μM ATP, 100 μM UDP-MurNAc-l-Ala–d-Glu and 4% DMSO (enzyme reaction) at 37 °C. The compounds were dissolved in DMSO at concentrations of 100, 500 and 1000 μM. The reaction was initiated by the addition of 1 mM *m*-DAP. After 30 min, 12.5 μL of gold-lock reagent with accelerator was added to terminate the reaction. After 5 min, a stabiliser was added and the reaction was left for 30 min at room temperature (25 °C) for colour development. The assay was performed in a final volume of 50 μL in a half-area Costar microtitre plate (Applied Biosystems, Carlsbad, CA). Inorganic phosphate binds to the gold-lock reagent to form a complex with an absorption maximum of 635 nm. MurE activity was estimated through phosphate release by measuring the absorbance of the reaction mixtures at 635 nm after 30 min. Absorbance values were corrected for background absorbance of the reaction mixtures and for any non-enzymatic hydrolysis of ATP in the absence of the enzyme (control reaction). Percent inhibition was calculated using a negative control (0%) and enzyme reaction (100% activity). All assays were performed in triplicate.

## 3 Results

### 3.1 Structural elucidation of compounds

Examination of the ^13^C spectra of compound **1** revealed similarities with other hypercalin derivatives previously isolated from *H. calycinum*, and the NMR data were in good agreement with those of hypercalin B [20]. Compound **2** was obtained as a yellow oil whose molecular formula was C_17_H_18_O_3_ as established by high-resolution mass spectrometry showing an [M+H]^+^ ion at *m*/*z* 271.1396. Its IR spectrum indicated the presence of a carbonyl (1651 cm^−1^) and benzene ring (1614 and 1580 cm^−1^), which was confirmed by the ^13^C NMR data (Table 1). The ^13^C NMR spectra exhibited 17 signals, which by ^1^H NMR and ^13^C Dept-135 experiments corresponded to six quaternary carbons, seven methines, one methylene, two methyls and one methoxy group. Five aromatic hydrogens at *δ* 7.70 m, 7.48 m and 7.47 m in the ^1^H NMR spectrum (Table 1) indicated the presence of a monosubstituted benzene ring. The ^1^H and ^13^C resonances of this moiety were assigned by close inspection of the heteronuclear multiple quantum coherence (HMQC) spectrum as *δ* 7.70 (H-2′, 6′) with *δ* 125.4 (C-2′, 6′), *δ* 7.48 (H-3′, 5′) with *δ* 129.3 (C-5′) and *δ* 7.47 (H-4′) with *δ* 131.0 (C-4′). Careful analysis of the heteronuclear multiple bond coherence (HMBC) spectrum revealed that the aromatic singlet at *δ* 6.61 (H-5) showed a ^2^*J* correlation to the carbonyl at 181.4 (C-4), a quaternary carbon at 157.1 (C-2) and a ^3^*J* correlation to the quaternary carbons at 111.5 (C-5) and 131.1 (C-1′).

**Fig. 1:**
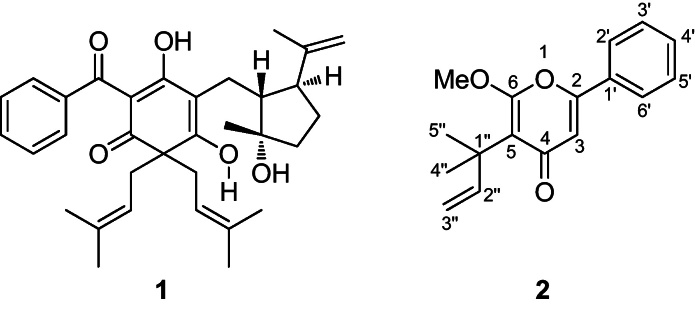
Structures of hypercalin B (**1**) and hyperenone A (**2**).

This indicated that the aromatic ring was attached to a benzene ring at C-1′. The signals at 4.90 (1H, dd, 13.5, 0.5 Hz) and 1.50 (6-H) were indicative of a 1,1-dimethylprop-2-enyl side chain in the molecule. The assignment of all ^1^H and ^13^C resonances as well as the moiety of the attachment of 1,1-dimethylprop-2-enyl side chain at C-2 was achieved by the HMBC experiment. In addition, a methoxy group at *δ* 4.06 demonstrated a ^3^*J* correlation to a quaternary carbon at 162.9 and confirmed its placement at C-3. These data together with analysis of the COSY spectrum revealed compound **2** to be hyperenone A (Fig. 1), which was previously isolated from *Hypericum mysorense* Heyne, but its ^13^C data were not reported [23]. Here we publish the ^13^C NMR data for hyperenone A for the first time based on extensive 1D- and 2D-NMR experiments (Table 1).

### 3.2 Effect of both compounds on *Staphylococcus aureus*

Compounds **1** and **2** were tested for their ability to inhibit the growth of *S*. *aureus* strains. Both of the compounds showed significant antibacterial activity, with a range of MIC values from 0.5 mg/L to 128 mg/L (Table 2).

### 3.3 Growth inhibition of *Mycobacterium* spp.

**Fig. 2:**
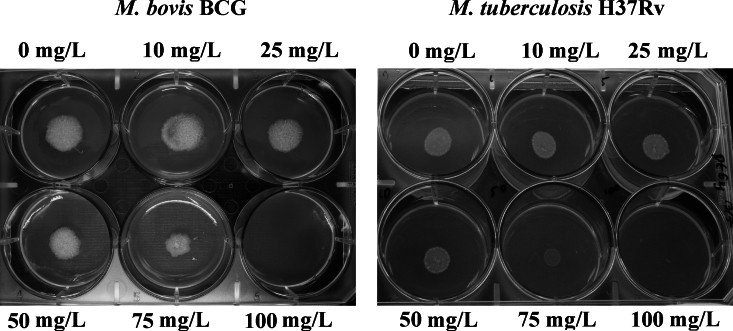
Effect of compound **2** on the growth of mycobacterial species using a spot culture growth inhibition assay. Approximately 500 *Mycobacterium bovis* BCG and *Mycobacterium tuberculosis* H37Rv cells were spotted on solidified Middlebrook 7H10 agar containing different concentrations of compound **2** in a six-well plate. Pictures were taken using a digital camera after 14 days of inoculation.

Both compounds were evaluated for their antibacterial activity against *M. tuberculosis* H37Rv and *M. bovis* BCG on solid agar media at different concentrations (10, 25, 50, 75 and 100 mg/L) in a six-well plate. Compound **1** was found to be inactive for both species, whilst compound **2** was active with MIC values >75 mg/L for both species. At 100 mg/L, compound **2** showed complete growth inhibition, whereas at 75 mg/L reduced growth was observed compared with the negative control (Fig. 2).

### 3.4 *Escherichia coli* JM109 killing curves

To evaluate whether the compounds’ selectivity affected slow-growing mycobacteria in comparison with fast-growing bacteria, whole-cell experiments were carried out. Comparative time measurement of *E. coli* growth after exposure to a high concentration of compounds, typically 200 mg/L, was determined by measuring the OD_600._ Compounds **1** and **2** were inactive against Gram-negative *E. coli* JM109.

### 3.5 Macrophage RAW 264.7 cytotoxicity

To determine whether the isolated compounds were toxic to mammalian cells, a cytotoxicity assay using RAW 264.7 cells at a serially diluted range of assay concentrations (3.125–200 mg/L) was performed and the cell viability was calculated. The result revealed that 100% eukaryotic cell viability was observed either up to 50 mg/L of compound **1** or up to 100 mg/L of compound **2** (Supplementary Fig. 1).

### 3.6 *Mycobacterium tuberculosis* MurE enzyme inhibition

**Fig. 3:**
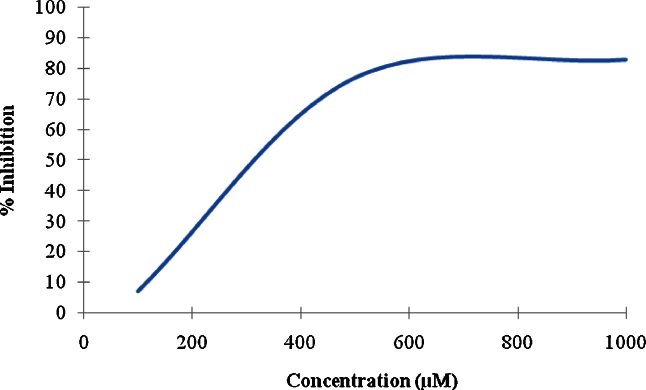
Effect of compound **2** on MurE enzyme activity. The *x*-axis represents the different concentrations of the compound tested in the assay and the *y*-axis represents the percent inhibition at different concentrations of the compound. The assay was performed in triplicate.

Inhibition of recombinant *M*. *tuberculosis* MurE was assayed under different concentrations of isolated compounds using the colorimetric detection of phosphate, which has been shown to be stoichiometrically coupled to *m*-DAP ligation [10,24]. Compound **1** did not inhibit MurE, whilst compound **2** showed 7%, 77% and 83% inhibition at 100, 500 and 1000 μM concentrations, respectively, having a 50% inhibitory concentration (IC_50_) of 320 μM (Fig. 3).

## 4 Discussion

The *Hypericum* genus is a valuable source of new phytochemicals and antibacterial compounds [13]. Compounds isolated from this group of plants have been shown to demonstrate antistaphylococcal activity [25,26] as well as many other biological activities such as antifungal properties [27]. Compound **1**, which was previously isolated from *H. calycinum*, exhibited in vitro growth inhibitory activity against the Co-115 human colon carcinoma cell line [20]. Compound **2** was previously isolated from *H. mysorense*. Here we show for the first time significant activity of these compounds with MIC values of 0.5–128 mg/L and 2–128 mg/L, respectively, against a panel of *S. aureus* strains, some of which are meticillin-resistant and multidrug-resistant (MDR) (Table 2). With the exception of EMRSA-15, compound **1** was slightly more active against all of the *S. aureus* strains compared with compound **2**. The strains included SA-1199B, a MDR strain that overexpresses the NorA efflux mechanism, the best characterised antibiotic pump in this species. SA-1199B also possesses a gyrase mutation that, in addition to NorA, confers a high level of resistance to certain fluoroquinolones. Against this strain, both compound **1** (MIC = 0.5 mg/L) and compound **2** (MIC = 2 mg/L) were more active than the control antibiotic norfloxacin (MIC = 32 mg/L). For MDR strain XU212, which possesses the Tet(K) efflux transporter and is resistant to both tetracycline and meticillin, compound **1** showed better inhibitory activity (MIC = 2 mg/L) compared with the positive control antibiotics (Table 2). Both compounds **1** and **2** showed moderate activity against a standard *S*. *aureus* strain (ATCC 25923) as well as RN4220, which possesses the MsrA macrolide efflux pump, but were less active than the control antibiotics. Compound **1** was slightly more active against EMRSA-16 compared with EMRSA-15, one of the major EMRSA strains occurring in UK hospitals. In addition, compound **2** (MIC = 2 mg/L) was marginally more active against SA-1199B than the fluoroquinolone ciprofloxacin control (MIC = 8 mg/L).

The in vitro antibacterial activity of compounds **1** and **2** did not inhibit the growth of *E. coli* and both were non-toxic to mammalian cells. Compound **2** was evaluated against the pathogenic *M. tuberculosis* H37Rv and the vaccine strain *M. bovis* BCG (Fig. 2) and was shown to be active at 100 mg/L for both species. Compounds with a higher degree of lipophilicity may play an important role in antimycobacterial activity. The mycobacterial cell wall contains lipophilic substances such as mycolic acid, thus lipophilic compounds would therefore have the advantage of better penetration through the cell wall to inhibit the growth of mycobacteria.

There is an ongoing effort to find inhibitors of Mur enzymes involved in peptidoglycan biosynthesis to target different bacterial diseases. Phosphonates, phosphinates and sulphonamides are amongst the inhibitors of MurC, MurD, MurE and MurF. Sova et al. [28] evaluated a series of phosphorylated hydroxyethylamines as a new type of small-molecule inhibitor of Mur ligases and found that the IC_50_ values of these inhibitors were in the micromolar range, making them a promising starting point for the development of multiple inhibitors of Mur ligases as potential antibacterial agents. Towards identifying the inhibitors of these enzymes, compounds extracted from *H. acmosepalum* were tested against MurE of *M. tuberculosis*. Of the two compounds tested, compound **2** showed a good dose-dependent inhibition of MurE enzyme activity. Although the precise antibacterial mode of action of compound **2** remains unknown, it is possible that its activity could be due to MurE enzyme inhibition. However, this hypothesis needs further investigation. Availability of the three-dimensional structure of MurE of *M. tuberculosis* facilitates further optimisation of this compound to improve its potency through fragment-based screening or docking studies [10,24]. This increases the chance of developing a natural product into a potential antimycobacterial compound with a novel mechanism of action.

To conclude, here we report the antibacterial activity of compounds **1** and **2** against a panel of *S. aureus*, *M. tuberculosis* H37Rv and *M. bovis* BCG bacteria. In addition, both of the compounds were non-cytotoxic to mammalian cells. Furthermore, compound **2** demonstrated specific dose-dependent inhibition of MurE enzyme activity of *M. tuberculosis*. As there is a lack of antibacterial compounds targeting Mur ligase enzymes, further research in this respect is required. The antibacterial activity of these compounds represents a promising starting point for further development.

## Figures and Tables

**Table 1:** Nuclear magnetic resonance (NMR) [500 MHz (^1^H) and 125 MHz (^13^C)] data for hyperenone-A (**2**) in CDCl_3_.

Position	^1^H *δ* ppm (*J*)	^13^C *δ* ppm
2		157.1
3		111.7
4		181.4
5	6.61	111.5
6		162.9
1		131.1
2′, 6′	7.70 m	125.4
3′, 5′	7.48 m	129.3
4′	7.47 m	131.0
1″		39.0
2″	6.25 dd (17.2, 10.4)	148.5
3″	4.90 d (17.2) 4.95 d (10.4)	108.5
4″/5″	1.50	27.7
MeO	4.06	56.2

**Table 2:** Minimum inhibitory concentrations (MICs) of compounds and control antibiotics against *Staphylococcus aureus* strains.

Agent	MIC (mg/L)					
	SA-1199B	EMRSA-15	EMRSA-16	RN4220	XU212	ATCC 25923
Compound **1**	0.5	128	64	16	2	16
Compound **2**	2	64	128	32	32	16
Norfloxacin	32	1	256	0.5	16	2
Tetracycline	0.25	0.25	0.25	0.25	64	0.25
Erythromycin	0.25	>128	>128	64	>128	0.25
Ciprofloxacin	8	8	16	0.25	1	0.25

## Appendix A Supplementary data

Supplementary Fig. 1 Bar diagram showing cytotoxicity of the compounds against mammalian cells (RAW 264.7 macrophages). DMSO, dimethyl sulphoxide; INH, isoniazid.
